# Detection of Liver Dysfunction in Severe Burn Injury with Bedside Measurement of Perfusion

**DOI:** 10.3390/medicina62030466

**Published:** 2026-02-28

**Authors:** Marianne Kruse, András Varga, Berthold Hoppe, Alexander Hoenning, Martin Aman, Klaus Hahnenkamp, Marc Dominik Schmittner, Volker Gebhardt

**Affiliations:** 1 Department of Anesthesiology, Intensive Care and Pain Medicine, BG Klinikum Unfallkrankenhaus Berlin, 12683 Berlin, Germany; andras.varga@ukb.de (A.V.); marc.schmittner@ukb.de (M.D.S.); volker.gebhardt@ukb.de (V.G.); 2 Department of Anesthesiology, University Medicine Greifswald, 17475 Greifswald, Germany; klaus.hahnenkamp@med.uni-greifswald.de; 3 Department of Laboratory Medicine, BG Klinikum Unfallkrankenhaus Berlin, 12683 Berlin, Germany; berthold.hoppe@ukb.de; 4 Health and Medical University, Campus Potsdam, 14471 Potsdam, Germany; 5 Biostatistics, Centre for Clinical Research, BG Klinikum Unfallkrankenhaus Berlin, 12683 Berlin, Germany; 6 Department of Hand-, Replantation- and Microsurgery, BG Klinikum Unfallkrankenhaus Berlin, 12683 Berlin, Germany; martin.aman@ukb.de; 7 Medical Faculty Mannheim of Heidelberg University, Ruprecht-Karls-University Heidelberg, 68167 Mannheim, Germany

**Keywords:** severe burn injury, liver insufficiency, organ failure, fluid resuscitation, burn shock

## Abstract

*Background and Objectives*: Severe burn injuries are still associated with high mortality. The length of intensive care stay is strongly influenced by the severity of organ failure, with multi-organ failure being the main cause of death in up to 40% of cases. Liver dysfunction is the second most common organ failure. Conventional diagnosis relies on static laboratory parameters that reflect damage already caused. Measuring the hepatic clearance of indocyanine green (LiMON^®^) offers a dynamic, bedside method for detecting liver dysfunction early, enabling timely therapy adjustments. *Materials and Methods*: In this prospective single-centre observational study, all patients admitted to the Unfallkrankenhaus Berlin Burns Centre from October 2022 to September 2024 with ≥30% TBSA burns were included. Liver function was assessed via LiMON^®^ within 24 h post-injury and every 48 h until day 14 or ICU discharge. Static liver parameters were measured in parallel. *Results*: We included a total of 23 patients. An initial measurement was only successful in 18 cases. On admission, six patients (33%) had normal liver function with a plasma duration rate (PDR) > 18% (PDR 30.9 ± 7.3%), while 12 (67%) showed reduced clearance (PDR 14.5 ± 2.6%). In 75% of cases (*n* = 9), function recovered within 48 h. Based on PDR progression, four liver function patterns were defined: “stable”, “recovery”, “late insufficiency”, and “failure”; a fifth pattern included all patients who were deceased during this study (“death”). These groups differed in fluid therapy, plasma transfusion, and catecholamines administered. PDR correlated well with aminotransferase levels. *Conclusions*: Dynamic liver function monitoring enables earlier detection of impairment than static markers. Early identification of at-risk patients could guide fluid management and improve outcomes. LiMON^®^ is a valuable tool in burn care, though alternative methods may be needed in patients with severe systemic hypoperfusion.

## 1. Introduction

Severe burns and the resulting burn disease continue to be life-threatening conditions associated with high mortality rates, especially in cases of extensive injuries and accompanying organ failure. Despite these risks, significant progress has been made in recent decades in acute care, intensive care and surgical treatment, which has contributed significantly to reducing mortality rates. Nevertheless, the often-lengthy intensive care treatment is physically and psychologically stressful for those affected and is associated with profound long-term health, psychosocial and economic consequences. Regaining quality of life is of central importance to patients and correlates negatively with the length of hospital stay [1]. Since the length of intensive care depends largely on the type and severity of organ failure, intensive care research must continue to focus on early detection, effective treatment and, in the best case, prevention of these complications.

After thermal injury with more than 15–20% body surface area affected, there is a pronounced direct loss of fluid and a generalised inflammatory response induced by the release of inflammatory mediators, leading to a “capillary leak” with loss of intravascular fluid as well as proteins into the interstitium. Hypothermia caused by insufficient thermal management and hypotension caused by inadequate fluid therapy can quickly lead to reduced blood flow to all organs and potentially result in multi-organ failure. To avoid complications and optimise therapy, early and continuous monitoring of organ dysfunction is necessary for early intensive care medicine during burn shock [1].

In up to 40% of cases, multi-organ failure is the cause of death in severely burnt patients, with liver failure being the second most frequent after kidney failure and occurring within the first two weeks [2,3]. While respiratory function can be closely monitored by pulse oximetric saturation and point-of-care blood gas analysis, and diuresis can be continuously recorded as a renal function parameter, a bedside tool for detecting the onset of liver dysfunction has so far been lacking in the routine intensive care of severely burnt patients.

In case of liver dysfunction or liver failure, the consequences for the further course of the disease and the patient’s outcome are fatal. The current standard diagnostic method for detecting liver dysfunction is the measurement of static parameters reflecting liver cell integrity and function (transaminases, cholestasis markers, and markers of liver synthesis). This method is more likely to detect liver damage that has already occurred than to accurately predict when the event is still fully reversible or preventable by suitable measures. Liver failure can be clearly defined using various scores [4,5], whereas the definition of liver insufficiency remains challenging.

The measurement of liver function via hepatic clearance of indocyanine green (LiMON^®^ Getinge AB, Gothenburg, Sweden) is already a known option in the surgical setting or in severe shock [6] to obtain a timely, non-invasive, bedside assessment of global liver function, thereby enabling earlier and more flexible therapy optimisation. This form of bedside testing is already used to evaluate liver function in the context of liver transplantation, as well as to predict the outcome of critically ill patients with sepsis, ARDS (acute respiratory distress syndrome [7], defined according to the Berlin criteria) and liver failure [8,9]. LiMON^®^ technology has also been used to show that increased intra-abdominal pressure, which is a common complication in severely burnt patients, is negatively correlated with liver function and can be used to improve prognostication [10]. As laboratory parameters alone cannot reliably classify mild to severe liver insufficiency, considering the combination could provide a better understanding of burn-associated liver dysfunction.

The measurement of liver function via hepatic clearance of indocyanine green in the context of burn shock is a method that has rarely been used to date. In a few publications, however, there are indications that the clinical progression of liver failure following burn shock can be represented both by LiMON^®^ measurements and by laboratory-detectable parameters [11]. Early detection of liver dysfunction with LiMON^®^ after severe burn injury is related to age, burn area and depth as well as cardiac function. Furthermore, it is associated with higher mortality if persistent [11]. In children, the ability to eliminate ICG as a measure of liver function depends on the age at the time of injury, any existing malnutrition and the presence of inhalation injury [12]. A further study of severely burnt adults with mainly deep burns >90% showed significantly worse liver function in the first two weeks in non-survivors [13].

The aim of the study was to utilise LiMON^®^ for early detection of liver dysfunction in severely burnt patients with a high risk of organ failure and to describe the course of liver function in comparison to the static parameters, as well as resuscitation management. To the best of our knowledge, this is the first time that a connection between volume management during burn shock and changes in liver function has been investigated.

## 2. Materials and Methods

After approval by the local ethics committee (Aerztekammer Berlin, 22/02/17, Eth-74/21) and national registration of the study (22/08/05, DRKS DRKS00027883), we conducted a monocentre prospective observational cohort study encompassing all patients who were admitted to the burn centre between October 2022 and September 2024 and met the inclusion criteria.

### 2.1. Patients

Inclusion criteria were the presence of a burn injury with a TBSA of >30% II–IV°, age ≥ 18 years and at least one debridement and hydrotherapy under general anaesthesia with mechanical ventilation. Exclusion criteria were age <18 years, pregnancy, allergy to iodine or indocyanine green, and hyperthyroidism or a change in therapy goal to best supportive care within the first 24 h. We also excluded patients with toxic epidermal necrolysis. Informed consent was obtained from the patients, their authorised representative or legal guardian.

After admission to the hospital and initial treatment in the emergency department, patients were transferred directly to the operating theatre of the burn centre. All patients received general anaesthesia, mechanical ventilation and a central venous catheter as well as an arterial catheter for invasive blood pressure measurement and enhanced haemodynamic monitoring (PulsioFlex^®^, Getinge, Gothenburg, Sweden). Fluid therapy was based on the Parkland formula. It was adapted to standard parameters such as heart rate, hourly urine output (HUO), base excess and lactate level, as well as parameters of enhanced haemodynamic monitoring (Cardiac Index—CI, Stroke Volume Variation—SVV and systemic vascular resistance index—SVRI). After initial surgical wound treatment, all patients underwent fibreoptic bronchoscopy to rule out inhalation injury and were transferred to the burn ICU. Dynamic measurement of liver function and blood sampling for static parameters (ALAT, ASAT, CHE, INR, GGT, Bilirubin) was performed within 24 h after trauma (T0) and repeated every 48 h until day 14 (T2.d2-T2.d14) and at the time of discharge from the ICU (T3); fluid requirements for resuscitation and plasma consumption were documented 24 h after trauma (T1).

We continued documenting clinical parameters and scores, used for the definition of organ failure (Table 1), for 14 d in total after trauma and at the time of leaving the intensive care unit.

The data were extracted from both patients’ electronic health records and the hospital information system Medico (Cerner Health Services^®^, Berlin, Germany) and the ICU data management system ICM (Dräger^®^, Lübeck, Germany). After importing the pseudonymised data to STATA (StataCorp LLC, College Station, TX, USA) version 16.1, incorrect data entries and measurement errors were identified through programmed plausibility checks and corrected accordingly.

### 2.2. Technic

LiMON^®^ is a technology for the non-invasive measurement of liver function and is considered a marker of splanchnic perfusion. Based on the elimination of the fluorescent dye indocyanine green (ICG), ICG can be detected and quantified after intravenous administration via its absorption and emission spectra using optical methods (non-invasive transcutaneous measurement).

The dye is eliminated exclusively through the liver after direct plasma protein binding. Therefore, the plasma disappearance rate of ICG (ICG-PDR) is an indicator of global liver function. Clearance is dependent on liver perfusion, liver cell function and biliary excretion. The diagnostic significance of the ICG-PDR as an indicator of liver function has been recognised for over 30 years and is used in many medical fields. Based on the summary of product characteristics, an ICG-PDR of 18–25% was considered normal, while an ICG-PDR of <18% was considered impaired liver function.

### 2.3. Statistics

Given the exploratory nature of this observational study, we did not conduct a formal sample size calculation. To draw meaningful conclusions from the statistical analyses [18,19], we targeted a sample size of 30 patients, with liver function measured via LiMON^®^.

In accordance with the Consolidated Standards of Reporting Trials (CONSORT) guidelines, data analyses were pre-specified a priori. Normal distribution was visually assessed using quantile-quantile (Q-Q) plots. Since a non-normal distribution was indicated, the correlation between dynamic and static liver function parameters was evaluated using Spearman’s rank correlation coefficient. The potential of dynamic measurement parameters to predict liver function was compared with static measurement parameters at later time points. The dichotomisation of the measurement parameters allowed the presentation of absolute and relative frequencies of correct and incorrect predictions.

Due to the lack of baseline perfusion measurements in five patients, we performed a sensitivity analysis to evaluate patients’ individual courses of plasma disappearance rates. Using the worst-case scenario, all missing values at baseline were considered as “poor perfusion” (worst-case) and compared with the results of the original data.

Depending on the scale level and distribution form of the data, absolute and relative frequencies, mean values or medians, and suitable measures of dispersion (i.e., standard deviations, ranges or interquartile ranges) are reported. Heat maps, line plots, and box plots were used to visualise correlations among variables, temporal trends in parameters, and differences between groups. Statistical analyses and graphical presentations were performed using STATA and R version 4.0.5 (R Foundation for Statistical Computing).

## 3. Results

We included 23 patients (male/female 14/9, mean age 47 ± 17 years, mean TBSA 47 ± 15%, mean Body mass index (BMI) 26.6 ± 4.5 kg/m^2^. All patients suffered from flame injury. Inhalation injury was detected in 12 patients (Table 2).

**Figure 1:**
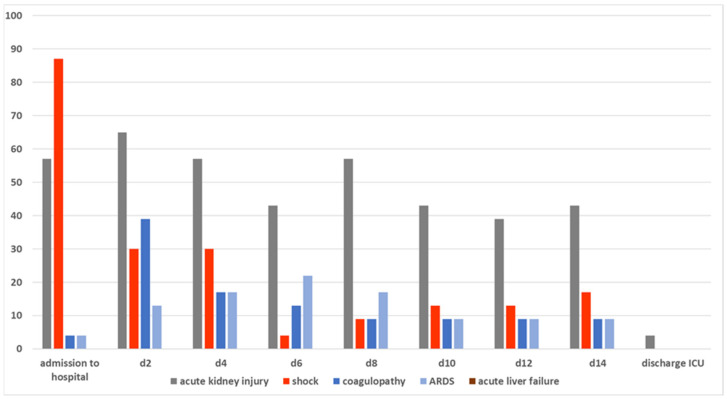
Organ failure during study time; *x*-axis Time points of measurement; *y*-axis share as percentage %.

Initial liver function measurements were successful in only 18 of 23 cases. In 5 patients, liver function could not be measured at the time of admission due to extremely limited perfusion in shock. Liver failure (King’s College criteria) did not occur (Figure 1).

For further group description, patients with missing ICG-PDR at T0 were excluded from the analysis. Dynamic measurements showed normal liver function on arrival in 6 cases (33%; PDR 30.9 ± 7.3%), while 12 (67%) showed impairment, with a 47% reduction in plasma clearance rate (PDR 14.5 ± 2.6%). Nine Patients (75%) recovered within 48 h. Considering the individual course of ICG-PDR, four groups of liver function were identified based on perfusion values, named “stable” (S), “recovery (R)”, “late insufficiency (L)”, “failure (F)” and a fifth group includes all patients who deceased while included in this study (“death (D)”) (Table 3).

Group allocation was based on global liver function (ICG-PDR >/< 18%) on arrival, after completion of resuscitation and during the long-term course of intensive care.

The assessment of group membership was solely clinical, based on observation of individual development of global liver function as reflected in PDR values.

**Figure 2:**
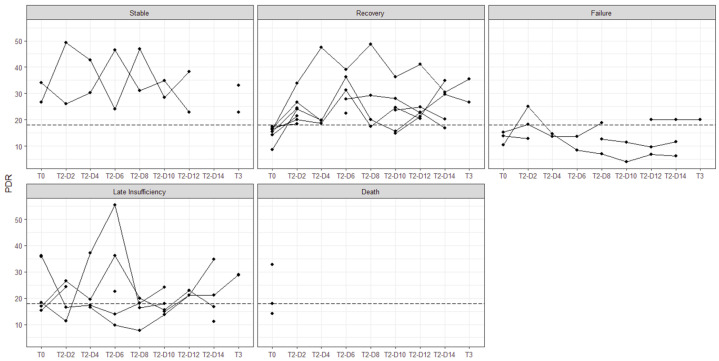
Different courses of liver function, *x*-axis: Time points of measurements. *y*-axis: Plasma disappearance rate. Normal liver function expected from PDR ≥ 18 (dotted line).

The individual development of liver function in the separate groups during the study period is shown in Figure 2.

**Figure 3:**
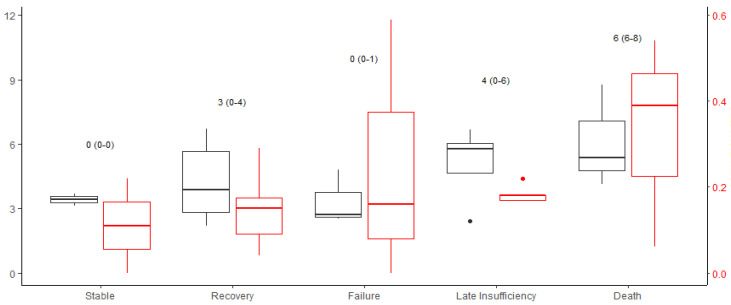
Burn shock therapy, *x*-axis: Groups; *y*-axis (black): fluid amount first 24 (mL/kgBW/%TBSA); *y*-axis (red): dosage of norepinephrine 24 h after trauma (µg/kg/min); annotation: number of FFP in 24 h (mean ± SD). Outliers are shown as dots.

Variations in resuscitation treatment were observed between the groups after 24 h, including differences in fluid therapy, transfusion of fresh frozen plasma, and catecholamine administration. These observed differences did not reach statistical significance and were identified in a small group (Table 4/Figure 3).

**Figure A1:**
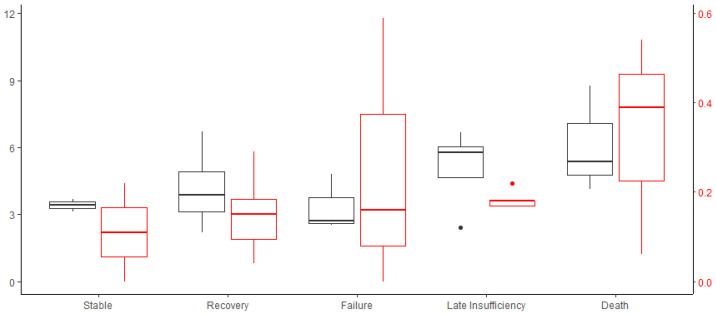
In burn shock therapy, missing PDR values at T0 are treated as poor for group classification (sensitivity analysis I). *x*-axis: Groups; *y*-axis (black): fluid amount first 24 (mL/kgBW/%TBSA); *y*-axis (red): dosage of norepinephrine 24 h after trauma (µg/kg/min). Outliers are shown as dots.

Worst-case analyses of the lack of measurement across five cases showed that T0 did not reveal relevant differences compared with the primary analysis (Appendix A, Figure A1).

The total fluid resuscitation volume in all patients over the first 24 h was 4.33 ± 1.7 mL/kg BW/% TBSA. Liver insufficiency could not be reliably assessed by classic laboratory parameters.

In the presentation of the absolute and relative frequencies of correct and incorrect predictions of the dynamic measurement, the sensitivity of the dynamic measurement indicating liver failure with static measurement parameters as a reference standard (ALAT, ASAT, CHE, INR, GGT, AP and total bilirubin) was less than 50% (Table 5).

**Figure 4:**
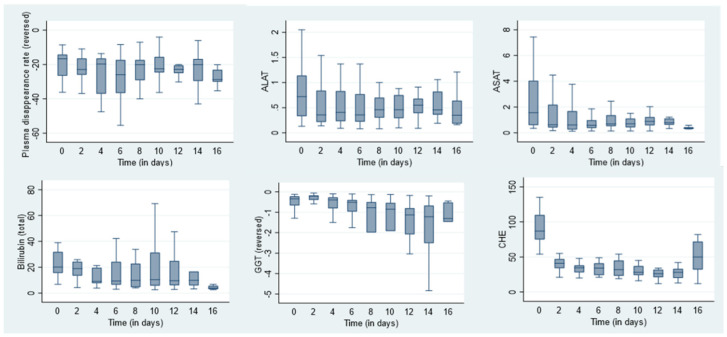
Course of static parameters over time points of measurement; *x*-axis: Time in days. *y*-axis: values of static parameters (ALAT/ASAT/GGT/CHE µkat/L; Bilirubin µmol/L).

Due to the lack of a clear definition of liver failure based on laboratory values, we performed a sub-analysis of static parameters in various combinations. The dynamic measurement’s sensitivity was also <50%. The course of the static parameters over the measurement time points is shown in Figure 4.

**Figure 5:**
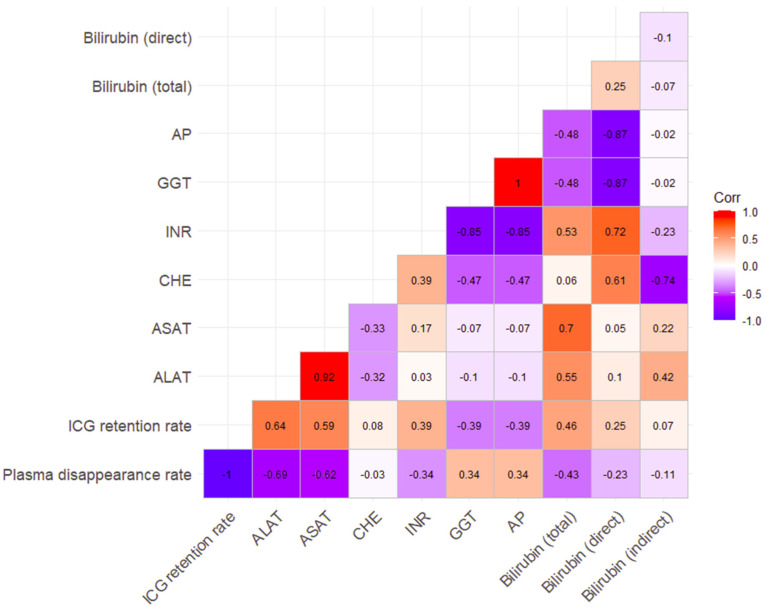
Heat map correlation, dynamic and static parameters, all time points. A total of nine patients (45%) died during hospital stay.

The correlation coefficient between dynamic and static measurement parameters, averaged across all subjects and separately by time of measurement, showed high correlations between both aminotransferases and ICG-PDR (ALAT r–0.69, ASAT r–0.62) and a moderate correlation between total bilirubin and ICG-PDR (r–0.43). The correlation between ICG-PDR and the parameters INR, GGT, AP and direct bilirubin was low; for indirect bilirubin and CHE, it was very low (Figure 5).

## 4. Discussion

While liver failure has a clear definition based on laboratory parameters [5,6], no classification is available for incipient liver dysfunction. Previous publications have used various combinations of parameters to describe deteriorating liver function [11,20]. The liver is one of the organs most sensitive to shock, with a high potential for recovery. Any deterioration in its condition should be detected as early as possible so treatment can be initiated.

Steinvall et al. described liver function with bedside ICG-PDR measurement in burn shock already in 2012. They found a lower incidence of early liver dysfunction (41%) than in our study. This may be because inclusion in our cohort required a minimum burn severity of 30% TBSA, and changes in liver perfusion correlate with burn severity [11]. The causes of liver dysfunction following a severe burn are various. Liver dysfunction following thermal injury is complex. Severe burns trigger a pronounced systemic inflammatory response, followed by the release of pro-inflammatory cytokines such as IL-1, IL-6, TNF-α, a metabolic stress response, the activation of immune cells, endothelial dysfunction and changes in microcirculation. Additionally, there is oedema and caspase-induced apoptosis of liver cells, compensated by an insufficient increase in hepatic cell proliferation. Clinical and experimental data have demonstrated that this response contributes to hepatocellular injury, metabolic dysregulation, and altered hepatic clearance early after burn injury, happening even if macrocirculation is restored. Therefore, burn-associated liver dysfunction should be understood as the result of an interplay between early perfusion deficits and inflammation-mediated mechanisms [21,22]. Even if hepatic changes after burn are multifactorial, hypoperfusion during shock is one of the known causes of liver damage [23]. It has been shown experimentally that thermal injury leads to a reduction in mesenteric blood flow of almost 60% within the first 4 h after burn [24] and alters liver perfusion [25]. As this is, in principle, a reversible condition, there are therapeutic options if detected in time. The initial measurement of liver function showed a severe impairment in a large proportion of patients at the time of hospitalisation. Through regular re-evaluation with LiMON^®^, we were able to recognise a deterioration or improvement at the bedside.

The decision to perform subgroup analyses despite the very small number of cases was driven by the heterogeneity of clinical courses observed in patients with comparable initial presentations and by the poor prognosis associated with the progression to liver failure. An important limitation of the present analysis is the small size of some subgroups, which limits statistical power and increases the risk of overinterpreting numerical differences. Although collapsing groups could increase statistical power, this approach was not pursued because it would reduce the interpretability of group-specific patterns. Consequently, the subgroup analyses should be regarded as exploratory and hypothesis-generating rather than confirmatory.

Although no statistically significant differences were detected, and the results should be interpreted cautiously, the findings provide clinically relevant observations that, when considered within a clinical framework, may help identify strategies to optimise resuscitation management and patient care. The trends observed can therefore only provide preliminary indications of possible underlying mechanisms and therapeutic approaches. A key focus of future studies should be to identify factors that contribute to the recovery or further deterioration of initially impaired liver function under treatment.

For this reason, volume therapy was one of the issues to be addressed in our investigation. However, the various clinical courses in our study suggest that other mechanisms also play a role in addition to volume therapy, especially if impairment occurs later. In the “recovery” group, liver function normalised within 24–48 h. Patients in this group tended to receive more fluids in the first 24 h than the group with no recovery of liver function (failure). It can be assumed that hypoperfusion and volume deficiency in burn shock had the greatest influence on liver function in these cases. The reasons for poorer liver function at later stages of the disease (group “late insufficiency”) are more difficult to identify and are most likely the result of multiple factors. Burn-associated sepsis already plays a major role at this stage [26,27], and burn-induced inflammation in combination with sepsis could be the cause of the late dysfunction [23,28]. There is evidence that over-resuscitation is associated with a higher rate of infectious complications [29]. In the group with late insufficiency, initial fluid levels tended to be higher than in the other groups, which may have contributed, in part, to deterioration in liver function over time.

Interestingly, the “stable” group received almost the same amount of resuscitation volume as the “failure” group. The explanation for this could be that due to burn shock, patients with pre-existing liver insufficiency have a higher volume requirement to compensate for the deficit, in contrast to patients with good liver function on arrival in the emergency room. So far, it is only an assumption that patients with pre-existing liver disease may have an increased fluid requirement and may benefit from an adaptation of the resuscitation strategy [30,31]. It is reasonable to surmise that this also applies to patients with reduced liver perfusion in shock and that inadequate volume therapy can lead to persistent insufficiency.

ICG elimination depends on hepatic blood flow, cellular function, and biliary excretion [8]. The sensitivity of the ICG-PDR measurement in our study for parameters related to cellular function and biliary excretion was low. Over the entire study period, the patients showed more abnormalities in static parameters than in functional measurements. In the absence of a scoring system for liver insufficiency regarding transaminases and parameters of biliary excretion or synthesis, it was difficult to define clear-cut-off values for the different parameters. It also remains unclear which combination of markers for the individual liver function areas can appropriately depict incipient liver dysfunction. Our data confirm the studies by Steinvall et al., in which it was also not possible to use static parameters reliably for the diagnosis of early-onset liver dysfunction or to demonstrate a correlation with dynamic measurements.

Static parameters, such as ALAT and ASAT, indicate cell damage or liver cell death that has already occurred, whereas dynamic measurements reflect actual liver function. Static values can only be detected in blood with a delay [20,23], so bedside monitoring of current liver function seems more practical, given the therapeutic possibilities in the context of a dynamic disease process. However, since the increase in transaminases most likely reflects a response to reduced liver perfusion [23], this may explain the high correlation between the ICG-PDR measurement and ALAT and ASAT. However, we agree that transaminases alone are insufficient to define functional liver insufficiency. We were unable to identify a clinically meaningful combination of additional laboratory parameters (e.g., INR, cholinesterase and bilirubin) that demonstrated a more consistent correlation with ICG-PDR. Therefore, no composite laboratory-based definition of liver dysfunction was proposed.

The definition of liver failure based on static laboratory parameters remains a challenge, as there are no established thresholds or validated parameter combinations for functional impairment prior to complete liver failure. The combination of dynamic measurements with selected laboratory parameters represents a possible approach for future characterisation of functional liver dysfunction before the onset of clinically manifest liver failure. This should be investigated in larger, prospective cohorts specialised in burns.

Alternative approaches used in other liver diseases include structural markers, such as measurement of liver stiffness or analysis of hepatic vein waveform, as well as haemodynamic parameters, such as the hepatic vein pressure gradient. These methods can provide information about chronic parenchymal changes and portal hypertension, but are not useful in the acute phase of burn shock. Imaging techniques such as liver volumetry or perfusion imaging [32,33], also assess morphological changes, hypertrophy or pre-existing circulatory disorders and are applicable when structural damage has already occurred. However, these techniques could be valuable in quantifying long-term or residual liver damage after severe burn trauma.

Apart from early detection, experimental approaches are addressing future treatment of liver dysfunction and incorporating regenerative and immunomodulatory strategies. Cell- and immune-based therapies, including mesenchymal stem cell- based approaches [34,35], have shown potential in experimental models of liver damage to improve hepatic microcirculation and reduce inflammation. This could be a future therapeutic option for the long-term consequences of thermal trauma on liver function.

## 5. Limitations

Due to the necessity of sufficient limb perfusion for adequate optical measurement of ICG emission, it was not possible to measure liver function in the initial phase in five cases, depending on the severity of burn shock. This led to a loss of data in a clinically very relevant phase of the disease. However, as bedside measurement of liver function could have an important influence on therapeutic decisions, alternative methods, such as liver function tests based on the metabolism of 13C-methacetin, may need to be considered as an alternative for severe burn shock.

Due to the small size and pronounced heterogeneity of the cohort, statistical significance tests could not be reliably performed. Despite this limitation, the observed differences between the groups suggest potential effects that should be further investigated in future studies with homogenised samples or larger sample sizes.

## 6. Conclusions

The rapid results of dynamic liver function measurements can enable intensivists to detect impairments even before the static parameters show the consequences of the cellular damage. The results should be interpreted with caution due to the limited group size and lack of statistical significance; however, from a clinical perspective, the findings suggest potential pathways for improving resuscitation management that merit further evaluation. It can be assumed that an adjustment in fluid management would benefit organ function; these patients should be identified at an early stage. As a proven method for assessing liver function in critically ill patients, this method is also useful for burn patients. However, in cases of severe shock and reduced overall perfusion, alternative methods of bedside liver function measurement should be considered.

## Figures and Tables

**Table 1:** Clinical parameters and scores used to define organ failure.

Organ Dysfunction/Failure	Score
liver	King’s-College-Criteria (INR/Serum Bilirubin/age/etiology) [4] ACLF Score [5]
lung	Berlin Criteria for acute respiratory distress syndrome (PaO_2_/FiO_2_, X-ray) [7]
kidney	Kidney Injury Score (AKI) (Serum creatinine/urinary output) [14]
shock	Norepinephrine > 0.1 µg/kg/min for MAP > 65 mmHg [15]
coagulopathy	INR > 1.5 and thrombocytes < 150 Gpt/L [16]
abdominal compartment	IAP > 20 mmHg for 24 h and organ dysfunction [17]

**Table 2:** Demographic data.

Number (%) or Mean ± SD	Study Group (*n* = 23)
Sex (%)	
female	9 (39.1)
male	14 (60.9)
Age (years)	47 ± 17
TBSA (%)	47 ± 15
Inhalation Injury	12 (52.2)
Only 2nd degree	2 (8.7)
Incl. 3rd degree (%)	18 (78.3)
Incl. 4th degree (%)	3 (13)
BMI (kg/m^2^)	26.6 ± 4.5
rBaux-Score	99 ± 24
ABSI-Score	9 ± 2

Legend: TBSA—Total body surface area; 2nd degree burn—partial-thickness burn, including epidermis and part of the dermis; 3th degree burn—full thickness burn including epidermis and dermis; 4th degree burn—including all skin layers, also muscles, tendons, bones; BMI—body mass index; rBaux-Score—revised Baux -Score, prognostic score, predicts burn caused mortality; ABSI Score—Abbreviated burn severity index, prognostic score, predicts burn caused mortality.

**Table 3:** Courses of liver function (n).

Stable (2)	patients with no restriction in liver function
Recovery (7)	patients with insufficiency on arrival and recovery in 24–48 h
Late	
insufficiency (5)	deterioration in liver function in the later course regardless of the initial phase (includes *n* = 2 from recovery group)
Failure (3)	consistently poor liver function without stable recovery
Death (3)	No new measurement possible due to death within the first 48 h

**Table 4:** Treatment in different groups.

Mean ± SD or Mean (Range)			
Group	Fluid Resuscitation	Catecholamine	FFP
	(mL/kgBW/%TBSA)	(µg/kg/min)	(n)
Stable	3.4 ± 0.4	0.11 ± 0.16	0 (0–0)
Recovery	4.3 ± 1.9	0.14 ± 0.08	3 (0–4)
Late insufficiency	5.1 ± 1.7	0.18 ± 0.21	0 (0–1)
Failure	3.3 ± 1.3	0.25 ± 0.31	4 (0–6)
Death	6.1 ± 2.4	0.33 ± 0.25	6 (6–8)

Legend: mL/kgBW/%TBSA—millilitre/kilogram bodyweight/total body surface area; µg/kg/min—microgram/kilogram/minute; FFP—fresh frozen plasma.

**Table 5:** Sensitivity of dynamic measurement—Total static parameters.

		Static Values Conspicuous		
		Yes	No	All
dynamic values				
conspicuous	yes	26	16	42
	no	35	67	102
	all	61	83	144

## Data Availability

The original contributions presented in this study are included in the article. Further inquiries can be directed to the corresponding author.

## Abbreviations

The following abbreviations are used in this manuscript:

2nd degree burn	Partial-thickness burn including epidermis and part of the dermis
3rd degree burn	Full thickness burn including epidermis and dermis
4th degree burn	Including all skin layers, also muscles, tendons, bones
µkat/L	microkatal/litre
µmol/L	micromol/litre
ABSI-Score	Abbreviated burn severity index, prognostic score, predicts burn caused mortality
ACLF	Acute on chronic liver failure
ACS	Abdominal compartment syndrome
AKI	Acute kidney injury
ALAT	Alanine Aminotransferase
ALF	Acute liver failure
AP	Alkaline Phosphatase
ARDS	Acute respiratory distress syndrome
ASAT	Aspartate Aminotransferase
BMI	Body mass index
CHE	Cholinesterase
GGT	Gamma-glutamyl transferase
ICG-PDR	Indocyanine green plasma disappearance rate
INR	International normalized ratio
kgBW	Kilogram bodyweight
rBaux	Revised Baux-Score, prognostic score, predicts burn caused mortality
TBSA	Total body surface area
